# Editorial: Orofacial Pain, Bruxism, and Sleep

**DOI:** 10.3389/fneur.2020.00555

**Published:** 2020-06-10

**Authors:** Mieszko Wieckiewicz, Efraim Winocur

**Affiliations:** ^1^Department of Experimental Dentistry, Wroclaw Medical University, Wrocław, Poland; ^2^Department of Oral Rehabilitation, The Maurice and Gabriela Goldschleger School of Dental Medicine, Tel Aviv University, Tel Aviv, Israel

**Keywords:** orofacial pain, sleep bruxism, awake bruxism, sleep, temporomandibular disorders

The task force on taxonomy of the International Association for the Study of Pain (IASP) defines pain as “An unpleasant sensory and emotional experience associated with actual or potential tissue damage, or described in terms of such damage” (1). Furthermore, IASP says that orofacial pain is a frequent form of pain perceived in the face and/or oral cavity. It may be caused by diseases or disorders of regional structures, dysfunction of the nervous system, or through referral from distant sources. The orofacial pain is a serious global problem which affects ~20% of human population each year. According to the current approach (2), bruxism is considered as two different behaviors observed during sleep and wakefulness, respectively, and the single definition for bruxism has been replaced by two separate definitions: (1) Sleep bruxism is a masticatory muscle activity during sleep that is characterized as rhythmic (phasic) or non-rhythmic (tonic) and is not a movement disorder or a sleep disorder in otherwise healthy individuals. (2) Awake bruxism is a masticatory muscle activity during wakefulness that is characterized by repetitive or sustained tooth contact and/or by bracing or thrusting of the mandible and is not a movement disorder in otherwise healthy individuals.

Even though the etiopathogenesis of bruxism is not fully understood, many different factors are believed to be associated with this muscular activity. An increasing number of evidence suggests a relationship between bruxism and other disorders, conditions or systemic diseases, including sleep breathing disorders, uncontrolled limbs movements during sleep, reflux disease, neurological disorders and orofacial pain (3, 4). This Research Topic aimed to determine the nature of sleep bruxism and its types, as well as investigate the relationship between sleep bruxism and orofacial pain as well as different general health diseases, conditions and disorders.

Nine articles had been included in this Research Topic.

Three published articles are concerning: the prevalence of orofacial and general pain related to muscular temporomandibular disorders (Kuć et al.), and prevalence of insomnia among patients with orofacial pain (Cruz et al.), and epidemiology of awake and sleep bruxism among adolscents (Winocur et al.).

Three articles are related to diagnostic methods in a group of people with increased activity of masticatory muscles (Osiewicz et al.; Szyszka-Sommerfeld et al.; Zani et al.). Furthermore, it should be emphasized that two from mentioned three papers describe the new diagnostic method of awake bruxism i.e., ecological momentary assessment and intervention principles for the study of awake bruxism behaviors. Next two papers assessed relationship between sleep bruxism and headache (Martynowicz et al.), as well as sleep bruxism and TMD-related pain (Smardz et al.).

A single article describes the nociceptive effect of platelet-rich plasma intramuscular injections in myofascial pain of masseter muscles (Nitecka-Buchta et al.).

Published articles confirm how difficult clinical issues are related to impaired function of the orofacial structures. At the same time, the described studies indicate that the etiology of complaints reported by patients can be very compound. Thus, it requires an objective diagnostic approach and personalized therapeutic strategies. Therefore, the approach to sufferers should be holistic based on broad evidence-based knowledge from various fields of medicine.

Editors strongly encourage potential readers to look at the articles.

## Author Contributions

All authors listed have made a substantial, direct and intellectual contribution to the work, and approved it for publication.

## Conflict of Interest

The authors declare that the research was conducted in the absence of any commercial or financial relationships that could be construed as a potential conflict of interest.

## References

[1] International Association for the Study of Pain (IASP) IASP Terminology. Available online at: https://www.iasp-pain.org/Education/Content.aspx?ItemNumber=1698#Pain

[2] Lobbezoo F Ahlberg J Raphael KG Wetselaar P Glaros AG Kato T. International consensus on the assessment of bruxism: report of a work in progress. J Oral Rehabil. (2018) 45:837–44. 10.1111/joor.12663 29926505 PMC6287494

[3] Khoury S Carra MC Huynh N Montplaisir J Lavigne GJ. Sleep bruxism-tooth grinding prevalence, characteristics and familial aggregation: a large cross-sectional survey and polysomnographic validation. Sleep. (2016) 39:2049–56. 10.5665/sleep.6242 27568807 PMC5070759

[4] Carra MC Huynh N Lavigne G. Sleep bruxism: a comprehensive overview for the dental clinician interested in sleep medicine. Dent Clin North Am. (2012) 56:387–413. 10.1016/j.cden.2012.01.003 22480810

